# Developing a digital transformation process in the manufacturing sector: Egyptian case study

**DOI:** 10.1007/s10257-022-00558-3

**Published:** 2022-06-22

**Authors:** Yasser Omar Abdallah, Essam Shehab, Ahmed Al-Ashaab

**Affiliations:** 1 Manufacturing Department, School of Aerospace, Transport & Manufacturing, Cranfield University, Cranfield, Bedford, UK; 2 Mechanical and Aerospace Engineering Department, School of Engineering and Digital Sciences, Nazarbayev University, Nur-Sultan, Kazakhstan; 3 Business Administration Department, Faculty of Commerce, Mansoura University, Mansoura, Egypt

**Keywords:** Digital transformation, Manufacturing, Digitalisation, Digital process, Multiple Criteria Decision Making (MCDM)

## Abstract

Digital transformation is of crucial importance in the manufacturing industry, especially after the COVID-19 pandemic because of the increasing need for remote working and socially distanced workplaces. However, there is a lack of a clear and well-defined process to implement digital transformation in manufacturing. This paper aims to identify the most critical stages to implementing digital transformation in the manufacturing sector. Twenty-one structured interviews with experienced specialists in digitalisation in the manufacturing sector in the Egyptian economy were held and used the Best–Worst Method to analyse the data as an analysis tool for a multiple criteria decision making (MCDM) approach. The digital transformation process comprises eight stages covering technology, management, communications, and customer elements. The main contribution of this work stage is the balance between the different elements of digital transformation—digital technologies, leadership and strategy, people and business processes—to create an integrated 8-step process of digital transformation in the manufacturing sector of developing economies such as the Egyptian economy.

## Introduction

With the exponential expansion of digital technologies and their widespread use in various sectors and industries, businesses are adapting their business models to achieve their ultimate objectives. This transformation has been accelerated by the coronavirus disease pandemic of 2019 (COVID-19) and its impact on the corporate sector and how customer needs are shaped (Müller et al. 2018).

This change has an impact on how businesses function both internally and externally connect with their consumers to deliver products and services. Manufacturing, being one of the most critical sectors of any economy, has been strongly impacted by the digitalisation of manufacturing processes through the development of new technologies that may be used to boost manufacturing operations productivity (Grabowska 2020).

In the manufacturing industry, digital transformation (DT) is vital for organisations to remain competitive since digitalisation of corporate operations is the only path to ensure sustainability in today’s competitive marketplaces (Mohelska and Sokolova 2018). This demonstrates the crucial significance of DT in enabling established firms to compete with native digital organisations that have arisen during the past 10 to 15 years. While academic research has highlighted DT in recent years, there is still debate regarding the term’s definition, context and implications, notably in the manufacturing industry (Bahlooq et al. 2020; Carolis et al. 2017; Ismagilova et al. 2019; Hartley and Sawaya 2019).

As DT is a very generic term, there is a lack of a well-defined definition of DT; moreover, those who attempt to explain DT frequently do so from their own perspectives and backgrounds (e.g., financial sector and government domain) (Culot et al. 2020). There is a lack of a clear and in-depth process of how the manufacturing industry can implement DT successfully. Because of this, most DT initiatives in the manufacturing industry fail due to a lack of understanding of the concept and requirements for implementing DT successfully.

To fill this gap, the present paper aims to propose and rank clear and well-defined stages for the manufacturing industry to implement DT successfully with validation of case studies from the Egyptian manufacturing sector as one of the most prominent developing economies. This will help any manufacturing organisation grasp the stages and procedures needed to commence the DT journey. The paper presents a literature review in developing DT process in the manufacturing sector and the main elements of DT implementation in the manufacturing industry, followed by the data collections methods and analysis. Finally, it demonstrates the research findings and formulates a DT implementation process in the manufacturing industry.

## Literature review

This section discusses a working definition of DT in the manufacturing industry. In addition, the main elements of DT that contribute to the success of any DT efforts are discussed to gain further insight into the stages needed to develop a clear DT process in the manufacturing industry.

According to the literature, few publications specifically define DT, particularly in the context of manufacturing, and definitions of DT and other related concepts, such as Industry 4.0 or digital disruption, often contradict (Mariani and Borghi 2019).

After conducting a study on the idea and analysing various definitions, a conceptual working definition of DT with a focus on the manufacturing industry has been developed (Abdallah et al. 2021a):

Digital transformation is a customer-centric mechanism that enables continuous improvement in the productivity of the manufacturing processes using advanced digital technologies, such as cloud computing, the Internet of Things (IoT), big data analytics, digital twins and artificial intelligence, in all aspects of the organisation.

While this field of research has lately gained prominence in academia, there is a dearth of evaluations of industrial organisations’ readiness to conduct DT procedures. Additionally, existing models in the literature place a heavy emphasis on Industry 4.0 technologies, with just a few models emphasising DT as a comprehensive overview of the process (Jones et al. 2021; Brozzi et al. 2020; Chirumalla 2021).

The scientific community and consultancy businesses are both heavily involved in DT research. The theoretical and methodological underpinnings of strategy selection, on the other hand, are relatively unclear. Typically, the theoretical grounds for choosing a DT method are addressed for specific domains. Additionally, larger organisations have made more significant advances in DT than smaller ones (de Jesus and Lima 2020). This is due to the substantial financial resources needed to incorporate digital technologies in all aspects of the organisation.

**Fig. 1:**
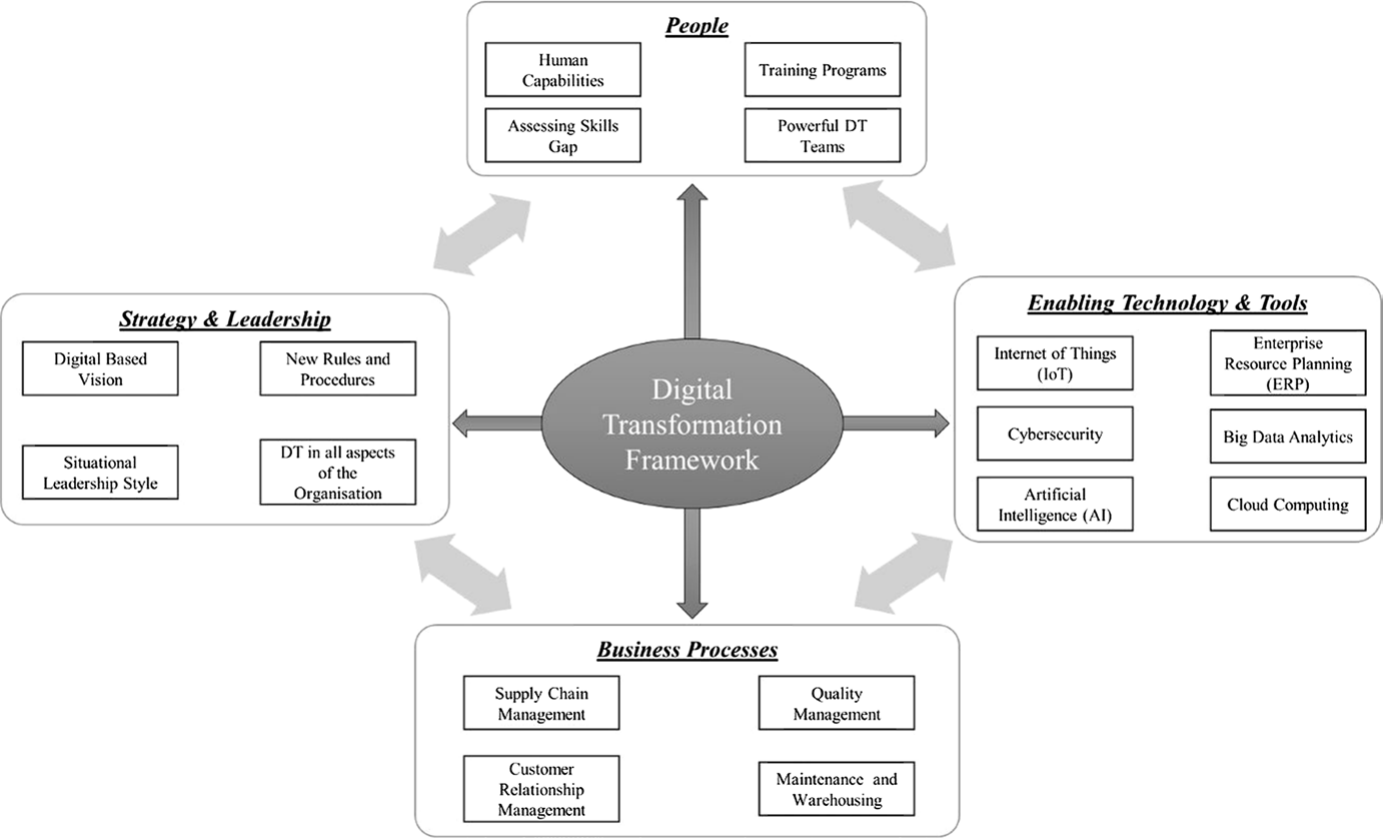
Digital transformation framework (Abdallah et al. 2021b)

Although there is research efforts on digital transformation in recent years, few suggested a comprehensive list of factors that could contribute to the successful implementation of DT, especially in the manufacturing sector (Paryanto et al. 2020; Zaoui and Souissi 2020). That is why in an initial phase of this ongoing research, the main elements that contribute to the success of DT processes in the manufacturing industry were listed. These are *people*, *leadership and strategy*, *enabling technologies and tools* and *business processes* (Abdallah et al. 2021b). As Fig. 1 shows, DT is not only about technical capabilities and infrastructure in the organisation; it should involve changing the organisational strategy and direction (Jones et al. 2021), capacity building activities towards digitalisation for the human factor and integrating all of the stakeholders around the organisation into it (Mihardjo et al. 2019; Oberer and Erkollar 2018).

Human capital is the foundation of any organisational change endeavour. DT necessitates the development of specialised capabilities in an organisation’s employees and workers (Paryanto et al. 2020; Dhanpat et al. 2020; Giang et al. 2021). The first stage in improving an organisation’s human capital is identifying the gap in digital capabilities in the organisation. Team building and management are also important aspects of human capital. DT is a collaborative effort that necessitates the integration of several departments and pieces to produce strong DT results. Typically a DT team includes (a) the digital transformation leader, who controls the whole process and resolves any conflicts that arise during a project’s execution; (b) the change agent, who encourages other members to adapt to change and be flexible in their job duties and work methods; (c) a business specialist with experience in marketing and distribution channel operations to provide insight into the project; (d) the data architect, who analyses and provides reports to assist senior management in making timely decisions, as this is a fast-paced process that necessitates flexibility and making the correct judgments at the appropriate time; (e) the financial analyst, who does the project’s cost–benefit analysis and manages the project’s financial budget; and (f) the UX specialist, who can reflect the customers’ voice and make the solutions provided by the organisation user-friendly. These team members work together to integrate DT efforts successfully (Chirumalla 2021; Jafari-Sadeghi et al. 2021).

As previously stated, the suggested definition of DT is a continuous process that must be integrated into an organisation’s strategy. Top management should foster an organisational culture that promotes DT activities and provide internal incentives to encourage employees to participate (Ismagilova et al. 2019).

The role of the leader is critical in DT. The most appropriate DT project is changing their leadership style to fit the scenario (Situational Leadership Style) (Tekic and Koroteev 2019). To be effective in the process, a leader needs to possess the following characteristics:

- Flexibility: Being open to change necessitates an entrepreneurial mindset.
- Diversified knowledge: This is acquired by seeing what is going on in other industries and determining what is working and relevant to their own.
- Priority and results focus: These are must-win processes that define success or failure and are focused on increasing the company’s market performance.
- Ownership and responsibility: People value bravery and accountability. To effectively lead, leaders and managers must hold themselves accountable for the overall performance of their teams (Krishnan et al. 2021).

Digital technologies are a crucial factor in the success of the DT process. Many studies mention various technologies employed in the DT process based on the operations field of an organisation as well as the technological infrastructure that existed in the economy, and all of these Industry 4.0 technologies are connected together to produce a more efficient DT process.

Artificial intelligence (AI) and its numerous applications are among the most important technologies that allow DT within any organisation. With AI and machine learning, machinery becomes more efficient and effective in its operations, as the machine itself has a better understanding of the processes and how to self-learn and fix any problems that arise within the manufacturing processes (Mittal 2020).

Businesses may use IoT technology to connect the physical and digital worlds. It enables manufacturing organisations to collect more data from machines and equipment, which aids in understanding production issues and how to address them more efficiently and run more productive operations (Olsen and Tomlin 2020).

Furthermore, one of the critical components in the effective implementation of DT is the security of the organisation’s digital world. With the rising number of cyberattacks occurring every day, cybersecurity technology has become critical in every organisation (Radanliev et al. 2020). Manufacturing companies must establish a robust cyber system to make data more accessible while also making it safe enough to withstand digital attacks.

Cloud computing is also a cornerstone of DT. With agile applications and the requirement to access data at any time and from any location, an organisation needs a strong cloud system that allows workers to interact with it and access the data they need to accomplish their operations (Butt 2020a). Machine intelligence and IoT generate massive volumes of data on everything that occurs in a factory. Big data analytics can give amazing insights into this data and how to gather, analyse and organise large volumes of data to assist management in making timely choices and providing new ways of innovation.

Robots are one of the modern technologies employed inside manufacturing organisations to undertake complicated duties to prevent worker fatigue and improve workplace health and safety standards (Bongomin et al. 2020). However, an industrial organisation must maintain the proper balance between robots and human labour to ensure the agility of the processes and make the most of the firm’s machines.

Enterprise Resource Planning (ERP) is a software toolbox that allows an organisation to connect all of its divisions to move information across large distances in real-time (Moeuf et al. 2018). Cloud ERP with a strong cloud system is more efficient in terms of data privacy and data transmission velocity than traditional ERP software.

These, among other examples of digital technologies, are the backbone of any DT initiatives in the manufacturing industry. Any organisation should choose the right combination of digital technologies suitable for its needs and financial capabilities (Mittal et al. 2018).

Connected business processes are critical, from supply chain integration with suppliers and their many layers to warehousing and quality control to customer relationship management and listening to consumer input in the DT process. Linking all activities across the value chain is critical because single initiatives will fail unless other processes from various departments are integrated (Butt 2020a).

This complexity of suppliers should be controlled collaboratively to deliver and store raw materials using supplier relationship management software utilising IoT and big data analytics to ensure that raw materials for industrial processes are not in short supply (Götz and Jankowska 2020). As a result, this must be meticulously monitored and coordinated with the procurement department.

Using sophisticated DT technologies, such as robots/cobots via advanced robotics management, in an organisation’s production process will increase productivity and allow the organisation to expand its products and services constantly.

A comprehensive literature review indicated that little effort was made in developing a balanced process for implementing DT in the manufacturing sector that focuses on DT's four dimensions. The major limitations of the existing DT processes is that it is primarily focused on adopting digital technologies. They also lacked how to overcome the challenges of implementing DT process resulting from human resistance to change and the development of communication plans for the integration between all stakeholders.

To overcome the above drawbacks, this research project has developed an integrated digital transformation process for the manufacturing industry. The developed process has taken into consideration all the aspects of the entire digital transformation process.

## Methodology

**Fig. 2:**
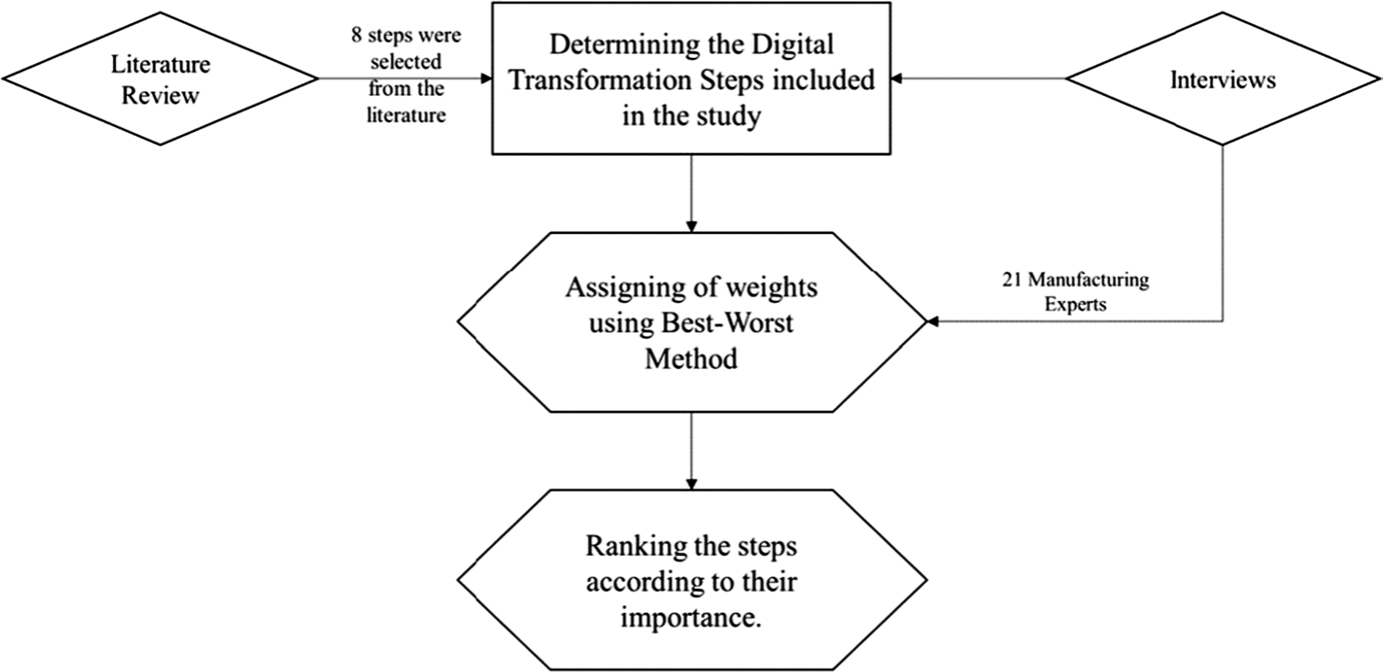
Methodology adopted for the study

This study followed an integrated approach to determining the stages necessary for DT in Egypt’s manufacturing industry, which included stakeholder interviews and literature reviews. The quantitative approach was taken by assigning weights to the pre-identified stages under each criterion using the best–worst multi-criteria decision-making method. Figure 2 illustrates the adopted research methodology.

Multi-criteria decision-making (MCDM) is a well-established technique for resolving complex situations, especially when several factors affect an objective (Wankhede and Vinodh 2020). It is a subset of decision theory. MCDM issues can be classified into two types according to their solution space, namely discrete and continuous. Multi-attribute decision-making (MADM) approaches can be used to handle discrete problems, whereas multi-objective decision-making (MODM) methods can be used to tackle continuous problems. MCDM has been used in a variety of ways over the years, including the Analytic Hierarchy Process (AHP), Technique for Order of Preference by Similarity to Ideal Solution, Analytic Network Process, and Preference Ranking Organization Method for Enrichment Evaluations, among others (Rezaei 2015).

The best–worst method (BWM) was adopted in this research. According to Rezaei (2015), the BWM is another effective MCDM tool that, due to its particular properties, may be utilised to study complex problems such as the one discussed in this paper: (a) the BWM can be used alone or in conjunction with other MCDM approaches; (b) the BWM’s consistency in comparisons makes it extremely trustworthy; (c) the BWM makes use of integers for convenience; and (4) in comparison to the AHP approach, the BWM approach requires fewer pairwise comparisons. This is why the BWM technique was used in this study to weigh the variety of aspects evaluated.

The BWM can be calculated using the following stages (Rezaei 2015):

1. The first step involves the creation of the decision criteria set: {*C*1*, C*2*, C*3*... Cn*}.
2. The second step involves the determination of the best (i.e., most important) and worst criteria (least important).
3. Step three is the determination of the favourite of the best criterion over every other criterion by assigning a number from 1 to 9. In this case, the vector for the resulting Best-to-Others would be as indicated in Eq. (1):
  1 $${\mathrm{A}}_{\mathrm{B}}=\left({\mathrm{a}}_{\mathrm{B}1},{\mathrm{a}}_{\mathrm{B}2},\dots ,{\mathrm{a}}_{\mathrm{Bn}}\right)$$
  where *aBj* denotes the preference of the best criterion *B* over the *j* criterion, and *aBB* = 1.
4. The next step is to determine the preference of the various criteria over the worst criterion by assigning any number between 1 and 9. In this instance, the vector for the Others-to-Worst would be as indicated in Eq. (2):
  2 $${\mathrm{A}}_{\mathrm{W}}={\left({\mathrm{a}}_{1\mathrm{W}},{\mathrm{a}}_{2\mathrm{W}},\dots ,{\mathrm{a}}_{\mathrm{nW}}\right)}^{\mathrm{T}}$$
  where the preference of criterion *j* over the worst criterion *w* is denoted by *ajW*, where *aWW* = 1.
5. The calculation of optimal weights are done at this step $\left({w}_{1}^{*},{w}_{2}^{*},\dots ,{w}_{n}^{*}\right)$. In assessing the optimal weight for the criteria (each pair of ${w}_{B}/{w}_{j}$ and *wj/ww*), we have ${w}_{B}/{w}_{j}={a}_{Bj}$ and ${w}_{j}/{w}_{w}={a}_{jw}$. To meet these conditions for the entire *j*, one must identify a solution in which the maximum absolute differences $\left|\frac{{w}_{B}}{{w}_{j}}-{a}_{Bj}\right|\text{ and }\left|\frac{{w}_{j}}{{w}_{w}}-{a}_{jw}\right|$ for entire *j* is minimised. This results in Eq. (3) when factoring the non-negativity and sum conditions for the weights.
  3 $$\begin{array}{*{20}l} {min\mathop {max}\limits_{j} \left\{ {\left| {\frac{{w_{B} }}{{w_{j} }} - a_{Bj} } \right|,\left| {\frac{{w_{j} }}{{w_{w} }} - a_{jw} } \right|} \right\}} \hfill \\ {{\text{s.t.}}} \hfill \\ {\sum\limits_{j} {w_{j} } = 1} \hfill \\ {w_{j} \ge 0, \quad {\text{for}} \,{\text{all}} \,\,j} \hfill \\ \end{array}$$
6. The optimal weights $\left({w}_{1}^{*},{w}_{2}^{*},\dots ,{w}_{n}^{*}\right)\text{ and }{\xi }^{*}$ would be obtained by solving Eq. (3). For the purposes of ensuring consistency, a consistency ratio (CR) also known as the *Ksi* value is assessed using ${\xi }^{*}$. The bigger the ${\xi }^{*}$ value, the less reliable the CR and comparisons become. The comparison becomes more reliable when the ${\xi }^{*}$ value is closer to zero.

The judgements for the analysis of the most critical stages in the manufacturing sector’s DT process were acquired from industry experts with a minimum of 10 years of experience in operations and production management roles and from different manufacturing sectors such as home appliance, food processing, clothing, pharmaceuticals and furniture. They were contacted via structured face-to-face interviews. In total, 28 experts were contacted, but only 21 responses were deemed worthy of consideration for the analysis following data cleaning. The rejected responses had not completely answered all of the questions to provide an accurate assessment of the interview.

## Results and data analysis

This section presents an overview of the most important stages in the DT process in the manufacturing industry, which cuts across all the sectors (i.e., managerial, economic, human and technical aspects). The quantitative results are also presented in this section.

### Quantitative analysis

The results of the BWM used to assign the various weights to the identified stages of the DT process in the manufacturing sector, as presented in Table 1, are discussed in this section. A total of eight stages were identified and ranked by experts. These stages shall be discussed in detail in subsequent sections in this study.

**Table 1:** Identified stages in the DT process

Identified stages in the DT process
Invest in the right technologies and tools (S1)
Involve all departments in developing a strategy (S2)
Invest in staff training (S3)
Draw up a comprehensive yet flexible/adaptable budget (S4)
Assign a board-level or C-level (Chief Executive Level) sponsor to the project (S5)
Pilot the project in one part of the business first (S6)
Communicate strategy and goals with employees (S7)
Communicate plans with customers (S8)

After assessing the various inputs from the consulted experts, *invest in the right technologies and tools* (S1) was selected as the most important step in the DT process, whereas *communicate plans with customers* (S8) was selected as the least important step in the DT process. As mentioned earlier in the introduction, all eight stages are crucial in the DT process, and in this paper, they are ranked according to their relative importance. In this example, S8 is least important compared to S1, but this does not mean it is not important.

**Fig. 3:**
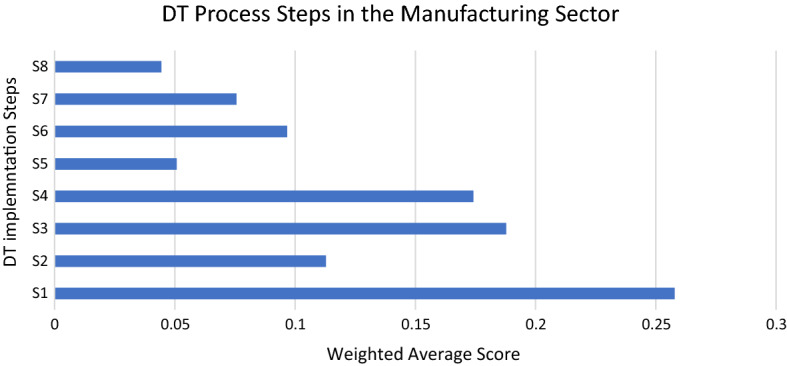
DT process steps in the manufacturing sector

Based on the preferences provided by the consulted stakeholders for each step as provided in “Appendix 1”, further calculations were done to ascertain the weights for the various factors. A Ksi* value (CR) of 0.079 was determined, close to zero. Remember that in the BWM, the closer the CR is to zero, the more consistent the experts' comparisons. The weights for the various stages are presented in Fig. 3. It reflects the respondents’ views on the various identified factors that are considered important stages in the DT process in the manufacturing industry and that have the potential to positively affect the development of DT efforts in the manufacturing industry in a developing economy such as the Egyptian economy.

According to the results, *invest in the right technologies* (S1) was assigned the highest weight of 25.8%, followed by *invest in staff training* (S3), which was assigned 18.8%. *Draw up a comprehensive yet flexible/adaptable budget* (S4) comes close to S3 with a weight of 17.4%. This reflects the importance of financial resources in the DT process in the manufacturing industry. *Involve all departments in developing a strategy* (S2) comes next with a weight of 11.3%, which reflects the connected-departments factor in the DT definition. This is followed by *pilot the project in one part of the business first* (S6) with a weight of 9.7%. Next comes *communicate strategy and goals with employees* (S7) with a weight of 7.6%. Communicating the strategy among employees reduces the resistance to change significantly, especially when employees feel that they are part of the decision-making process. The two least important stages are *assign a board-level or C-level sponsor to the project* (S5) with a weight of 5% and *communicate plans with customers* (S8) with approximately the same weight of 4.5%.

### Formulating DT process stages in the manufacturing industry

**Fig. 4:**
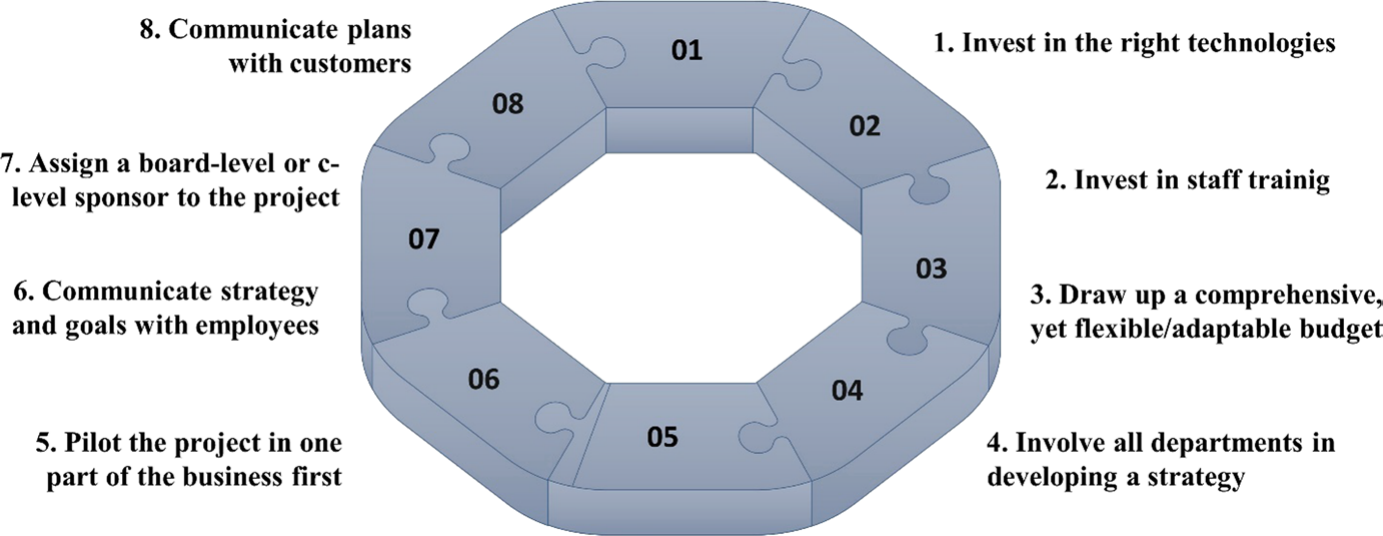
DT process in the manufacturing industry

This section will discuss the implementation stages and best practices of DT in the manufacturing sector after the readiness assessment of an organisation. Assessing the readiness of the organisation's capabilities helps the decision-maker get an overall insight into the DT processes needed for the organisation to move forward in the DT journey. As mentioned earlier, this process needs to be continuous and needs to be further updated regularly to cope with the advancement of digital technologies and the shift in customer needs and wants. Figure 4 summarises all the stages in the DT process ranked based on the BWM after conducting the interviews.

#### Invest in the right technologies and tools

The right understanding of these technologies and how the manufacturing organisation could use them properly in their daily activities is crucial to the success of any DT process. These digital technologies range from Artificial Intelligence (AI), Internet of Things (IoT), big data analytics, robots, and many other tools. Investing in the right technologies that suit an organisation’s structure and size will help the organisation best utilise these technologies to get the most return on investment in the shortest period of time (Benitez et al. 2020). This practice will differ from one manufacturing sector to another and will depend on many factors such as the organisation’s financial and human capabilities (Dalmarco et al. 2019). It is very important to emphasise investing in technologies that are right for the organisation, not the best technologies existing in the market, as these best technologies may not be suitable for the organisation.

#### Invest in staff training

**Fig. 5:**
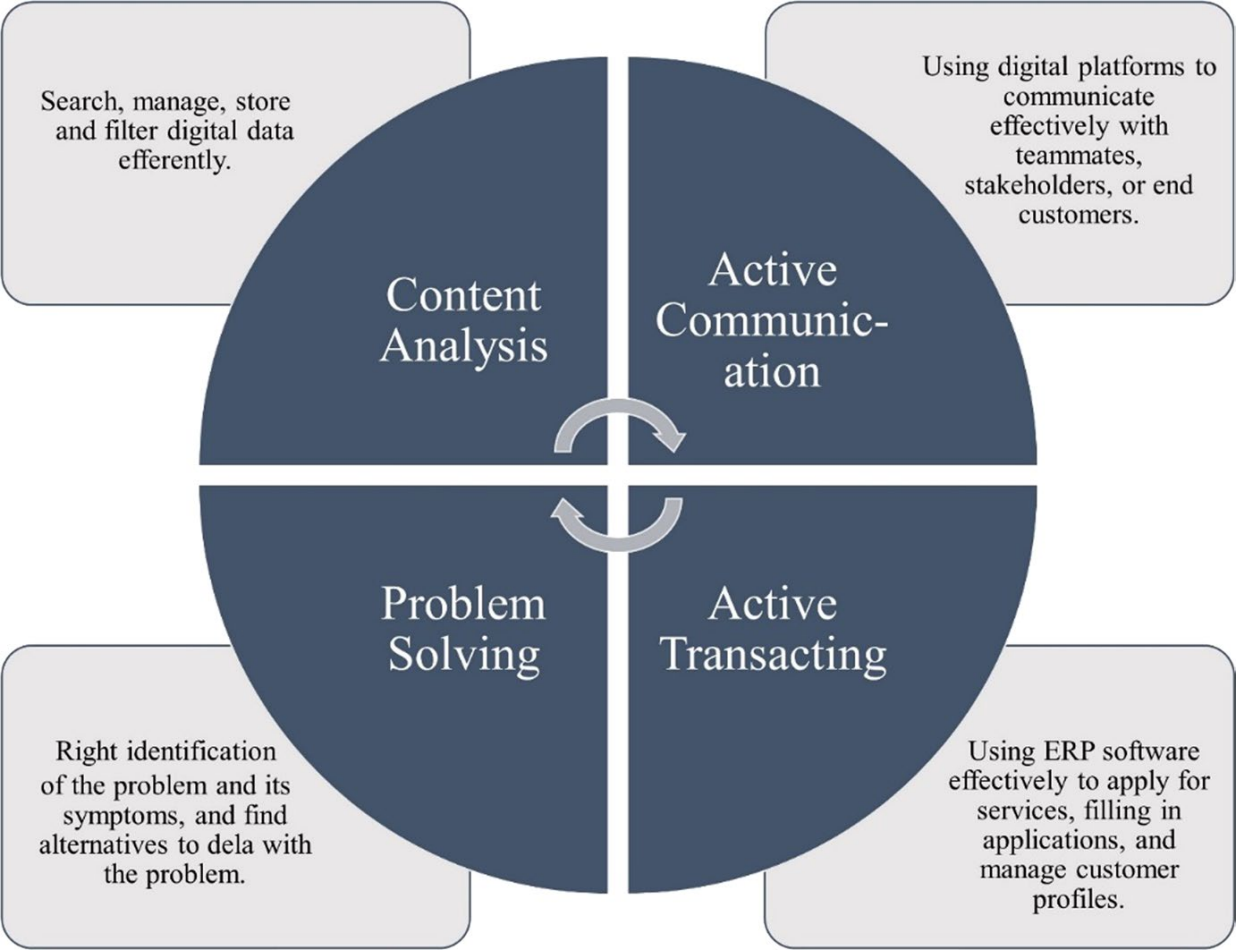
Essential digital skills for DT

One of the main challenges of DT is the lack of skills necessary to cope with this transformation, either intellectual skills or technical skills (Butt 2020b). This is due to a lack of training programs provided by an organisation to their employees as well as the demographic nature of the workforce in some economies. Investing in training to build technical skills to cope with the advancement of digital technologies is crucial to the success of the DT process. Figure 5 summarises the essential digital skills needed for implementing DT.

Not only are technical skills necessary, but intellectual and human skills are also very important in the DT process (Chirumalla 2021). Investing in training programs, such as innovation management or managing change, especially for middle managers, is of great importance to help managers understand the resistance of change for their employees and know ways to cope with this resistance and overcome it.

#### Draw up a comprehensive yet flexible/adaptable budget

Financial capabilities are one of the main criteria for a decision-maker to take into consideration when setting a DT strategy. Developing a budget that suits the needs of the manufacturing organisation to achieve its goals from DT is of great importance. This will also determine the level of digital technologies advancement that the organisation should invest in Ing et al. (2019). When setting a budget for DT, several factors need to be taken into consideration—for example, the cost–benefit analysis as well as the expected return of investment and the project payback period. Every manufacturing organisation should allocate a specified percentage of its profits to DT processes each year to cope with the developments of digital technologies, as this a continuous process.

#### Involve all departments in developing a strategy

As mentioned above, DT is not only about operation or production departments, but it should also happen across all departments of an organisation (Chirumalla 2021). When developing a strategy for DT, the decision-makers should involve all departments in the organisation as well as the stakeholders most affected by this change—for example, the suppliers. Many organisations go beyond that and develop a co-strategy with their main suppliers or main distributors to ensure that their DT efforts are well organised so they get the most out of this change (Ciano et al. 2021).

Involving all departments in developing the strategy will significantly reduce the resistance to change when implementing these change strategies, and this will enhance the efficiency of the processes and reduce any efforts to make this change fail (Horváth and Szabó 2019).

#### Pilot the project in one part of the business first

DT is a huge project in terms of scale and scope. Implementing the project in all the businesses inside an organisation in the first stages of the project would bear a great risk both financially and administratively. For this reason, one of the best tactics to initiate a DT project is to pilot it first on one the business units of the organisation. This pilot may take place in one of the production lines or one of the production processes inside the production line. The scale of the piloting will depend on the financial capabilities of the organisation as well as the skills already equipped in its labour. Moreover, it will depend on how far the organisation wants to reach in adapting DT projects and processes.

#### Communicate strategy and goals with employees

Communication is one of the most important procedures in any projects that aims to make significant change (Kovaitė et al. 2020). Top management should communicate the goals of implementing DT to the working labour of the organisation. This will help reduce any fear from the employees that digital technologies will eliminate their jobs and convince them that applying digital technologies will reshape their jobs, not replace them (Horváth and Szabó 2019). Another benefit from communication is that it opens up space to invite any innovative ideas that employees might have in implementing these kinds of projects. This will increase the sense of participation in forming this strategy and make them more committed to achieving the goals of this strategy.

#### Assign a board-level or C-level sponsor to the project

Top management support is one of the key success practices from previous experiences in manufacturing organisations (Krishnan et al. 2021). This support has a great influence on reshaping the organisational culture to be more digital-oriented and help the labour understand the significance of DT in their daily activities. This support also encourages middle managers to take initiative in this direction and showcase their digital processes within their departments to achieve a more digital-oriented workflow.

#### Communicate plans with customers

The final best practice is to communicate your plans with the customers. As the customers are the heart of any organisation, they should be aware of any change in the plans of a company, especially if it relates to the user experience or communication channels with the customers (Zaoui and Souissi 2020). For example, making the customer more aware of new chatbot feature on the organisation’s website and how to use this feature will maximise the use of this feature.

## Implications

### Theoretical implications

Although there is an extensive literature for DT implementation, few gave a detailed process for DT implementation in the manufacturing sector (Jafari-Sadeghi et al. 2021; Anderson and Ellerby 2018; Luthra et al. 2020; Stoianova et al. 2020; Balog and Knapčíková 2021). This research adds to the literature by developing a prioritised DT implementation process that is specifically tailored for the manufacturing industry using data collected from top experts in the manufacturing field in an evolving economy that is focused on the manufacturing industry like the Egyptian economy. Moreover, this research considers most of the aspects in the DT process implementation from technology to human factor and leadership support.

### Practical implications

The suggested model help manufacturing firms in the developing economy to formulate a comprehensive strategy to implement DT, especially for those manufacturing firms that are in the beginning of their digital transformation journey. By offering this process, manufacturing firms can understand and prioritise their business objectives to suit their capabilities in order to achieve a successful digital transformation. As stated earlier, digital transformation is not only a one-time process, rather, it is a continuous process that needs the integration of all aspects of the organisation. That is why organisation need to adapt their processes and prioritise them according the dynamic factors that can affect their digital transformation journey. That is why this suggested framework for a DT process offers the most important eight steps of the DT and the flexibility to prioritise them according to the manufacturing firm capabilities and their surrounding business environment.

## Conclusions

This paper aims to identify and determine the most important stages for DT implementation in the manufacturing sector after assessing the readiness of the manufacturing organisation to begin this process. After studying the literature review, we determined eight stages and ranked them according to their importance. Twenty-one structured interviews with manufacturing experts in the Egyptian economy were held and analysed using the BWM to rank the stages according to the interviewees’ perspectives. The stages in the DT process are as follows, according to importance: (1) invest in the right technologies and tools, (2) invest in staff training, (3) draw up a comprehensive yet flexible/adaptable budget, (4) involve all departments in developing a strategy, (5) pilot the project in one part of the business first, (6) communicate strategy and goals with employees, (7) assign a board-level or C-level sponsor to the project, and (8) communicate plans with customers. Understanding the dimensions and factors of each of these eight stages will help manufacturing organisations implement DT successfully, especially when they have yet to start their DT process.

This research has limitations. First, this study was applied to the Egyptian economy, which has developing economy, which has different characteristics from the developed economics, even in adopting different digital technologies. Second, the purpose of this research is to address the implementation phase of the DT process; other post-implementation phases may include measurement and control, feedback and improvement.

Future research may include applying the same process in a specific manufacturing industry and seeing how changing the manufacturing industry impacts the process. Other future research may include applying the same DT process in a developed economy and making a comparative analysis between both processes or applying the same process in a different sector and measuring the differences in the process.

## Appendix 1

See Table 2.

**Table 2:** Data analysis using the BWM

Stages	E1	E2	E3	E4	E5	E6	E7	E8	E9	E10	E11	E12
S1	0.184237	0.330425	0.188106	0.168798	0.295385	0.339223	0.330951	0.339223	0.297214	0.334495	0.198675	0.176619
S2	0.122825	0.087879	0.125404	0.112532	0.123077	0.141343	0.103903	0.141343	0.185759	0.10453	0.07947	0.117746
S3	0.286592	0.219697	0.310784	0.168798	0.123077	0.084806	0.207806	0.084806	0.074303	0.209059	0.13245	0.267452
S4	0.122825	0.146465	0.125404	0.2711	0.184615	0.212014	0.138538	0.212014	0.123839	0.069686	0.317881	0.176619
S5	0.073695	0.024606	0.027262	0.056266	0.027692	0.028269	0.059373	0.028269	0.092879	0.083624	0.02649	0.050463
S6	0.092119	0.073232	0.094053	0.112532	0.092308	0.070671	0.069269	0.070671	0.123839	0.083624	0.099338	0.117746
S7	0.092119	0.062771	0.075242	0.084399	0.092308	0.070671	0.059373	0.070671	0.074303	0.083624	0.07947	0.070648
S8	0.025589	0.054924	0.053745	0.025575	0.061538	0.053004	0.030786	0.053004	0.027864	0.031359	0.066225	0.022708

Stages	E13	E14	E15	E16	E17	E18	E19	E20	E21	Avg	Rank
S1	0.312695	0.210383	0.189949	0.189003	0.31068	0.194175	0.18782	0.31773	0.31773	0.257786	1
S2	0.130895	0.105191	0.094974	0.075601	0.097087	0.097087	0.125213	0.099291	0.099291	0.112878	4
S3	0.196343	0.140255	0.126632	0.303551	0.12945	0.31068	0.301651	0.132388	0.132388	0.18776	2
S4	0.130895	0.325137	0.126632	0.126002	0.194175	0.12945	0.125213	0.198582	0.198582	0.174079	3
S5	0.029088	0.070128	0.047487	0.075601	0.029126	0.029126	0.075128	0.066194	0.066194	0.050808	7
S6	0.065448	0.028689	0.308666	0.126002	0.07767	0.097087	0.028458	0.099291	0.099291	0.096667	5
S7	0.078537	0.060109	0.075979	0.075601	0.097087	0.07767	0.09391	0.056738	0.056738	0.075618	6
S8	0.056098	0.060109	0.029679	0.028637	0.064725	0.064725	0.062607	0.029787	0.029787	0.044404	8

## References

[CR1] Abdallah YO, Shehab E, Al-Ashaab A (2021). Understanding digital transformation in the manufacturing industry: a systematic literature review and future trends. Prod Manag Dev.

[CR2] Abdallah YO, Shehab E, Al-Ashaab A (2021). Towards managing digital transformation in manufacturing industry: theoretical framework. Adv Transdiscipl Eng.

[CR3] Anderson C, Ellerby W (2018) Digital maturity model. Deloitte, no. February, pp 9–12

[CR4] Bahlooq SA, Omar MA, Mezher T (2020) Analyzing the United Arab Emirates manufacturing sector and its readiness for Industry 4.0. In: 2020 IEEE international conference on technology management, operations and decisions, ICTMOD 2020. 10.1109/ICTMOD49425.2020.9380611

[CR5] Balog M, Knapčíková L (2021). Advances of intelligent techniques used in Industry 4.0: proposals and testing. Wirel Netw.

[CR6] Benitez GB, Ayala NF, Frank AG (2020). Industry 4.0 innovation ecosystems: an evolutionary perspective on value cocreation. Int J Prod Econ.

[CR7] Bongomin O, Gilibrays Ocen G, Oyondi Nganyi E, Musinguzi A, Omara T (2020). Exponential disruptive technologies and the required skills of Industry 4.0. J Eng (united Kingdom).

[CR8] Brozzi R, Riedl M, Matta D (2020). Key readiness indicators to assess the digital level of manufacturing SMEs. Procedia CIRP.

[CR9] Butt J (2020). A strategic roadmap for the manufacturing industry to implement Industry 4.0. Designs.

[CR10] Butt J (2020). A conceptual framework to support digital transformation in manufacturing using an integrated business process management approach. Designs.

[CR11] Chirumalla K (2021). Building digitally-enabled process innovation in the process Industries: a dynamic capabilities approach. Technovation.

[CR12] Ciano MP, Dallasega P, Orzes G, Rossi T (2021). One-to-one relationships between Industry 4.0 technologies and lean production techniques: a multiple case study. Int J Prod Res.

[CR13] Culot G, Nassimbeni G, Orzes G, Sartor M (2020). Behind the definition of Industry 4.0: analysis and open questions. Int J Prod Econ.

[CR14] Dalmarco G, Ramalho FR, Barros AC, Soares AL (2019). Providing Industry 4.0 technologies: the case of a production technology cluster. J High Technol Manag Res.

[CR15] De Carolis A, Macchi M, Negri E, Terzi S (2017). A maturity model for assessing the digital readiness of manufacturing companies. IFIP Adv Inf Commun Technol.

[CR16] de Jesus C, Lima RM (2020). Literature search of key factors for the development of generic and specific maturity models for Industry 4.0. Appl Sci.

[CR17] Dhanpat N, Buthelezi ZP, Joe MR, Maphela TV, Shongwe N (2020). Industry 4.0: the role of human resource professionals. SA J Hum Resour Manag.

[CR18] Giang NTH, Hai PTT, Tu NTT, Tan PX (2021). Exploring the readiness for digital transformation in a higher education institution towards industrial revolution 4.0. Int J Eng Pedagog.

[CR19] Götz M, Jankowska B (2020). Adoption of Industry 4.0 technologies and company competitiveness: case studies from a post-transition economy. Foresight STI Gov.

[CR20] Grabowska S (2020). Smart factories in the age of Industry 4.0. Manag Syst Prod Eng.

[CR21] Hartley JL, Sawaya WJ (2019). Tortoise, not the hare: digital transformation of supply chain business processes. Bus Horiz.

[CR22] Horváth D, Szabó RZ (2019). Driving forces and barriers of Industry 4.0: do multinational and small and medium-sized companies have equal opportunities?. Technol Forecast Soc Change.

[CR23] Ing TS, Lee TC, Chan SW, Alipal J, Hamid NA (2019). An overview of the rising challenges in implementing Industry 4.0. Int J Supply Chain Manag.

[CR24] Ismagilova LA, Gileva TA, Galimova MP, Sitnikova LV, Gilev GA (2019) The digital transformation trajectory of industrial enterprises. In: Proceedings of the 33rd international business information management association conference, IBIMA 2019: education excellence and innovation management through vision 2020, pp 2033–2045

[CR25] Jafari-Sadeghi V, Garcia-Perez A, Candelo E, Couturier J (2021). Exploring the impact of digital transformation on technology entrepreneurship and technological market expansion: the role of technology readiness, exploration and exploitation. J Bus Res.

[CR26] Jones MD, Hutcheson S, Camba JD (2021). Past, present, and future barriers to digital transformation in manufacturing: a review. J Manuf Syst.

[CR27] Kovaitė K, Šūmakaris P, Stankevičienė J (2020). Digital communication channels in Industry 4.0 implementation: the role of internal communication [Digitalni komunikacijski kanali u implementaciji industrije 4.0: Uloga interne komunikacije]. Manag.

[CR28] Krishnan S, Gupta S, Kaliyan M, Kumar V, Garza-Reyes JA (2021). Assessing the key enablers for Industry 4.0 adoption using MICMAC analysis: a case study. Int J Product Perform Manag.

[CR29] Luthra S, Kumar A, Zavadskas EK, Mangla SK, Garza-Reyes JA (2020). Industry 4.0 as an enabler of sustainability diffusion in supply chain: an analysis of influential strength of drivers in an emerging economy. Int J Prod Res.

[CR30] Mariani M, Borghi M (2019). Industry 4.0: a bibliometric review of its managerial intellectual structure and potential evolution in the service industries. Technol Forecast Soc Change.

[CR31] Mihardjo LWW, Sasmoko S, Alamsjah F, Elidjen E (2019). Digital leadership role in developing business model innovation and customer experience orientation in Industry 4.0. Manag Sci Lett.

[CR32] Mittal S, Romero D, Wuest T (2018). Towards a smart manufacturing toolkit for SMEs. IFIP Adv Inf Commun Technol.

[CR33] Mittal P (2020) Impact of digital capabilities and technology skills on effectiveness of Government in public services. In: 2020 International conference on data analytics for business and industry: way towards a sustainable economy, ICDABI 2020. 10.1109/ICDABI51230.2020.9325647

[CR34] Moeuf A, Pellerin R, Lamouri S, Tamayo-Giraldo S, Barbaray R (2018). The industrial management of SMEs in the era of Industry 4.0. Int J Prod Res.

[CR35] Mohelska H, Sokolova M (2018). Management approaches for Industry 4.0—the organizational culture perspective. Technol Econ Dev Econ.

[CR36] Müller JM, Kiel D, Voigt KI (2018). What drives the implementation of Industry 4.0? The role of opportunities and challenges in the context of sustainability. Sustainability.

[CR37] Oberer B, Erkollar A (2018). Leadership 4.0: digital leaders in the age of Industry 4.0. Int J Organ Leadersh.

[CR38] Olsen TL, Tomlin B (2020). Industry 4.0: opportunities and challenges for operations management. Manuf Serv Oper Manag.

[CR39] Paryanto P, Indrawan H, Cahyo N, Simaremare A, Aisyah S (2020) Challenges toward Industry 4.0: a case study of power plants in Indonesia. In: Proceeding—2nd international conference on technology and policy in energy and electric power, ICT-PEP 2020, vol 3, pp 272–276. 10.1109/ICT-PEP50916.2020.9249918.

[CR40] Radanliev P (2020). Cyber risk at the edge: current and future trends on cyber risk analytics and artificial intelligence in the industrial internet of things and Industry 4.0 supply chains. Cybersecurity.

[CR41] Rezaei J (2015). Best-worst multi-criteria decision-making method. Omega (united Kingdom).

[CR42] Stoianova O, Lezina T, Ivanova V (2020). Corporate culture: impact on companies.

[CR43] Tekic Z, Koroteev D (2019). From disruptively digital to proudly analog: a holistic typology of digital transformation strategies. Bus Horiz.

[CR44] Wankhede VA, Vinodh S (2021). Analysis of Industry 4.0 challenges using best worst method: a case study. Comput Ind Eng.

[CR45] Zaoui F, Souissi N (2020). Roadmap for digital transformation: a literature review. Procedia Comput Sci.

